# Intestinal Microbiota Correction in the Treatment and Prevention of Urinary Tract Infection

**DOI:** 10.5152/tud.2022.22119

**Published:** 2022-11-01

**Authors:** Nikolay V. Sturov, Sergey V. Popov, Vladimir A. Zhukov, Tatiana V. Lyapunova, Ekaterina I. Rusanova, Georgy N. Kobylyanu

**Affiliations:** 1 General Medical Practice Department, RUDN University (Peoples’ Friendship University of Russia), Moscow, Russian Federation; 2 Medical Informatics and Telemedicine Department, RUDN University (Peoples’ Friendship University of Russia), Moscow, Russian Federation

**Keywords:** Intestinal microbiota, dysbiosis, urinary tract infections, probiotics

## Abstract

Intestinal microbiota is a topical subject of modern research. The maintenance of a healthy intestinal microbiota is an important component of homeostasis, and violations of its composition and functions, called dysbiosis, are associated with a number of diseases, including urinary tract infections. Antimicrobial therapy leads to significant changes in the intestinal microbiota and causes the possibility of urinary tract infection recurrence. In this regard, it is important to study methods of microbiota correction in order to restore its structural and functional integrity.

**Main Points**

- Current research suggests a link between intestinal dysbiosis and urinary tract infections.
- Consideration should be given to the impact of treatment of urinary tract infections on the intestinal microbiota.
- There are various approaches to correcting the intestinal microbiota, showing different results in the treatment and prevention of urinary tract infections.
- Maintaining a healthy intestinal microbiota and correcting dysbiosis may be a promising method for the treatment and prevention of urinary tract infections and requires further research.

## Introduction

The intestinal microbiota (IM) is a complex ecological system of bacteria and other microorganisms that inhabit the human intestine and are in dynamic balance and interaction both among themselves and in the “microorganism–host” system. Thanks to the active study of IM in recent years, it has become known about the important role of its normal state in maintaining human health. The relationship of IM disorders, called dysbiosis, with certain disease development and an increase in human susceptibility to infections has also been determined.^1-8^ The IM is the largest and crucial microbial community of the human body, which is regarded as a “regulator” of the microbiota of other body areas, and, presumably, as a source of origin of such local communities, including the microbial communities in the urinary tract, also called “urobiome.”^9-13^

### Intestinal Dysbiosis and Urinary Tract Infections

Intestinal dysbiosis can be described by means of the following components: active growth of opportunistic and pathogenic microorganisms that can cause diseases or have a pro-inflammatory potential, a decrease in commensals that perform a number of useful functions, including those with an anti-inflammatory effect, as well as a decrease in microbial diversity.^14-18^ It was found that such IM disorders can be one of the key factors in the development of urinary tract infections (UTIs).^19^ It is known that most UTI pathogens originate from IM and that uropathogenic bacteria are able to form so-called “reservoirs” in the intestine. The mechanism of pathogen translocation from the intestine with subsequent contamination of the perineum, periurethral space, and penetration into the urinary tract is an important part of the pathogenesis of uncomplicated UTI.^20^ The results of a number of studies confirm that intestinal dysbiosis is a predisposing factor for further UTI development.^21-23^ The significance of increased opportunistic microbiota representatives in the intestine was studied in the work of Magruder et al^21^: the authors demonstrated that an increase in the relative number of *Escherichia* and *Enterococcus* in the intestine is related to the future development of the corresponding bacteriuria type, as well as UTI (for *Escherichia). *The genetic similarity established by metagenomic sequencing between the strains isolated from the urine and feces of patients gives additional value to the study. In another study by Magruder et al.^24^ it was found that the high relative number of 2 bacterial taxa, *Faecalibacterium* and *Romboutsia*, may be related to a reduced risk of *Enterobacteriaceae* bacteriuria and UTI in kidney transplant recipients. An inverse relationship between the relative number of the above 2 taxa and *Enterobacteriaceae* is also reported. The results of this work indicate that the IM normal state and sufficient commensal presence are related to a reduced risk of developing UTI. Paalanne et al^23^ also noted a higher content of *Enterobacteriaceae* in the IM of pediatric patients with UTI in comparison with healthy ones. In turn, representatives of *Peptostreptococcaceae* were found to be more common in healthy children than with UTIs. In the work of Piteková et al.^22^ an increased *Escherichia* *coli *number up to 9-10 lg CFU/g and a decrease in* Bifidobacterium *spp. and *Lactobacillus *spp. titers up to 6 (5.0; 8.0) and 5 (4.0; 6.0) lg CFU/g, respectively, was found in patients with UTI. A high frequency and titers of other *Enterobacteriaceae* family members—*Klebsiella *spp.,* Enterobacter *spp.,* Proteus* spp.—were noted. The significance of the commensal microbiota is in the fact that it contributes to intestinal resistance to colonization by various pathogens. This is achieved through competition for nutrients, adhesion sites, as well as through the production of antimicrobial metabolites and changes in the pH of the medium.^17,18^ Considering that the pathogenic strain urovirulence is not determined by a specific genetic structure and also that such gene expression is of great importance, the commensal IM part can be considered as one of the key factors in pathogen virulence suppression.^18,25^ It is also known about the relationship between the decrease in IM diversity and UTI development. Patients with recurrent UTI cases have a less diverse microbiota that produces insufficient amounts of butyrate, which performs a number of important functions and is necessary to maintain homeostasis and intestinal barrier integrity. Worby et al^26^ found a decrease in the butyrate-producing *Faecalibacterium, Akkermansia, Blautia,* and *Eubacterium hallii* presence in the IM of patients with recurrent UTI, which is largely similar to the microbiota state after exposure to antibiotics. It is reported that there was no significant difference in the average relative number of *E.* *coli* in the control groups and groups with UTIs. At the same time, the patient susceptibility to UTI in the corresponding group cannot be reliably explained by the presence of more virulent pathogen strains because strain carrier patterns and phylogroup distribution were generally similar to healthy individuals. It is assumed that the development of infection may be facilitated by dysbiosis, due to which the uropathogenic strain gene expression becomes more accessible. Indeed, a higher concentration of short-chain fatty acids in a healthy microbiota is able to suppress *E. coli *virulence factors, including pronounced inhibition of bacterial motility.^26,27^

Thus, each of the considered intestinal dysbiosis components is directly related to UTI development, and correction of IM changes may be a promising method in the strategy of therapy and this disease prevention.

### Impact of Antimicrobial Therapy for Urinary Tract Infections on the Intestinal Microbiota

The mainstay of UTI treatment is antimicrobial therapy (Table 1). Despite proven antibiotic efficacy in the treatment of UTIs and a pronounced effect on the microbiota, this approach is not considered in the context of IM correction and restoration. Moreover, there is evidence of an increase in the likelihood of infection recurrence, as well as antibiotic resistance development with the use of antibiotics.^47,48^ A systematic review showed a dramatic decrease in microbiota diversity after antibiotic therapy.^47^ Violations in the IM composition can persist for a long time—up to 6 months or more after treatment discontinuation. There was a significant decrease in *Bifidobacterium* spp. diversity when using doxycycline, as well as a decrease in the enterobacteria, *Bifidobacterium* spp., and *Lactobacillus* population when using clarithromycin. Phenoxymethylpenicillin, nitrofurantoin, and amoxicillin had the least effect on the IM.^49^ In the above-mentioned work of Magruder et al.^24^ the use of antibiotics was also associated with a decrease in the relative *Faecalibacterium,* *Romboutsia* number, which confirms the negative effect of antimicrobial therapy on the IM and its connection to the intestinal dysbiosis development and incidence of UTIs. Mulder et al^50^ when studying the IM of 1413 people found the strongest and most lasting effect on microbiota diversity of macrolides and lincosamides—the decrease in beta diversity persisted up to 4 years, and with the use of beta-lactams and quinolones—up to 1 year. The use of drugs with high anaerobic activity was associated with an increase in *Firmicutes*, and the use of antimicrobials without the activity was associated with *Bacteroidetes.* It should also be noted the importance of insufficiently effective pathogen eradication from the urinary tract, when uropathogenic strains persist in the intestine, despite antibiotic treatment. Such an effect of antibiotics entails intestinal dysbiosis progression, and hence the risk of UTIs.^26^ Thus, antimicrobial therapy significantly changes the IM, stimulates the virulence factor expression by uropathogens, and increases the risk of future UTIs.^51,52^ Considering all of the above, non-antimicrobial methods of treatment and prevention may be of particular interest in this context (Table 2).

### Importance of Probiotics for Intestinal Microbiota Correction

Among the methods for correcting the IM, much attention is paid to probiotics. Probiotics are live microorganisms that, when administered in adequate amounts, confer a health benefit on the host.^64^ Probiotic strains, which have a number of positive effects, are used to competitively displace pathogens from the intestinal environment and restore the IM.^18,65^ The use of probiotics takes into account their potential to reduce the prevalence of antibiotic-resistant microorganisms due to the widespread use of antimicrobials.^66^ The inclusion of probiotics in therapy strategies for various diseases is under active consideration. A combination of probiotics and prebiotics has been shown to be effective on the day of liver transplantation, which subsequently reduces the infection rate after surgery. Reduced length of stay in the intensive care unit and at the hospital and reduced duration of antibiotic use have been reported, which highlights the promise of using probiotics in fighting antibiotic resistance prevalence.^67^ The ability of probiotic strains to suppress uropathogens was demonstrated by Shim et al^68^: lactobacilli (*Lactobacillus gasseri, Lactobacillus rhamnosus, Lactobacillus acidophilus, Lactobacillus plantarum, Lactobacillus paracasei, Lactobacillus acidophilus*) showed inhibitory activity against uropathogens (*E. coli* [ESBL](−), *E. coli* [ESBL](+)*, Proteus vulgaris, Enterococcus fecalis*) with an average inhibitory zone of 10.5-20.0 mm in diameter. Numerous studies focus on the effect of probiotics and prebiotics on the pathogenic bacteria’s adhesive ability.^69^ There is evidence of strain-specific inhibition of uropathogenic bacteria adhesion to bladder cells by *Lactobacillus* spp. probiotic (*Lactobacillus salivarius* *UCM572*, *L. plantarum* *CLC17*, and *L acidophilus 01*).^70^ Prebiotics can be used to stimulate beneficial bacteria growth. For example, insoluble dietary fiber from soy hulls promoted the proliferation and increased adhesion time of *L. plantarum* and *Bifidobacterium longum.* ^71^ The ability of *L. plantarum* and *L. rhamnosus* strains to displace biofilms of *E. coli *and *Staphylococcus aureus* from medical-grade silicone has been determined.^72^ Bacterial biofilms are a big problem for therapy and very important for infectious agents. They allow pathogens to greatly enhance antimicrobial resistance, making eradication much more difficult.^73^

In a systematic review, based on an analysis of 16 studies with a total of 1426 participants, it was shown that the use of probiotics in therapy is more effective than the use of placebo in reducing the rate of UTI recurrence (risk ratio (RR): 0.52; 95% CI, 0.29-0.94).^74^ In a major study, Beerepoot et al^75^ compared trimethoprim-sulfamethoxazole use (480 mg once daily) with twice daily oral administration of *L. rhamnosus* GR-1 and* Lactobacillus reuteri* RC-14 for 12 months in 252 women with recurrent UTIs. In both groups, there was a decrease in the frequency of UTI episodes: from 7.0 to 2.9 episodes per year in the antibiotic group and from 6.8 to 3.3 in the probiotic group. An important feature of the probiotic use was the absence of an increase in antibiotic *E. coli *resistance in the group. Lee et al^76^ demonstrated a reduction in the recurrence of UTIs with probiotic prophylaxis for 6 months. The rate of recurrence was 8.2%, compared with 20.6% in the no prophylaxis group (*P* = .035) and was not significantly different from 10.0% in the antibiotic (trimethoprim/sulfamethoxazole) group (*P* = .532). The antibiotic susceptibility profile in the probiotic prophylaxis group was significantly better. Wolff et al^77^ found that oral probiotic administration (*L. rhamnosus *GR-1 and *L. reuteri *RC-14) did not significantly change the uropathogen to lactobacillus ratio (U/L) in the urine of four participants compared to placebo. It is important to note that the probiotic strains may have had an impact by changing the IM. One of the features of probiotic use for the treatment of various diseases is that their activity and effectiveness may depend on the specific strain.^78^ This circumstance makes it necessary to evaluate the effectiveness and safety of each strain.^79^ Studies analyzing the effectiveness of probiotics in the treatment and prevention of UTIs are heterogeneous. Considerable heterogeneity is observed, including variability in populations evaluated, strains, dosage, and treatment duration.^18^ Thus, the use of probiotics in patients with UTI remains a matter of debate so far. Large-scale, high-quality research is required to definitively determine the importance of probiotics in UTI prevention and treatment.

### Role of Fecal Microbiota Transplantation in the Correction of Intestinal Dysbiosis

Fecal microbiota transplantation (FMT) from healthy donors to recipients is a rather effective method of IM correction. There is evidence of several clinical cases of FMT application in patients with a history of recurrent UTI. Aira et al^80^ reported that after FMT regarding *Clostridioides difficile* infection in a 93-year-old patient with recurrent UTI caused by *E. coli* and *Pseudomonas aeruginosa*, no new symptomatic episodes of UTI were diagnosed in monitoring during 1 year. The state of the patient’s IM before FMT can be described as dysbiosis with a predominance (74.23% out of relative number) of *Enterobacteriaceae* (in particular*, Klebsiella *spp.), whose proportion significantly decreased (to 0.07%) after FMT. After the FMT, IM diversity has also increased. The donor microbiota was dominated by the *Bacteroidaceae* family (phylum *Bacteroidetes*) and the *Lachnospiraceae* and *Ruminoccoccaceae* families (phylum *Firmicutes*), which corresponds to modern data on the normal IM. *Clostridium difficile* infection is the main indication of FMT. There is an association of this disease with UTI: The basis of UTI therapy is the use of broad-spectrum antibiotics, which in turn is a major risk factor for the development of an infection caused by *C. difficile.* A retrospective analysis of patient therapy for *C. difficile *infection showed a significant reduction in the incidence rate of UTIs, on average, from four episodes to one episode per year after FMT (*P* = .01).^81^ An effective FMT was reported in a 50-year-old woman with 8 culture-positive (predominantly *E. coli*, including ESBL-producing ones) episodes of UTIs within the last 2 years. As a result, when examining the urinary microbiota, there was a gradual decrease in *Enterobacteriaceae* during the follow-up period from 8.3% at the beginning of the study to 0.5% on the 84th day, while the culture of all the subsequent urine samples (on the 14th, 39th, and 84th days) remained negative. After 9 months of follow-up, the patient showed no symptoms of UTI.^82^ Similarly, for 12 months, symptoms were absent after FMT in a patient suffering from recurrent UTIs due to ESBL-producing *Klebsiella pneumoniae*. When examining the urinary and fecal microbiota after FMT, no *K. pneumoniae* was detected.^83^

### Influence of Diet on the iIntestinal Microbiota State

Diet is one of the most significant and modifiable factors influencing the state of the IM. The possibilities of dietary interventions to correct IM are widely known. Nowadays, discussions are underway regarding the possibilities of using the diet influence on the IM structure and functions in the treatment of various diseases.^84^ Indigestible dietary fiber, such as fiber, has a pronounced positive effect on microbiota improvement. A systematic review and meta-analysis based on an analysis of 64 studies involving 2099 people showed that dietary fiber supplementation increases the number of *Bifidobacterium *spp. (standardized mean difference (SMD): 0.64; 95% CI, 0.42, 0.86; *P* < .00001) and *Lactobacillus *spp. (SMD: 0.22; 95% CI, 0.03, 0.41; *P* = .02), as well as butyrate concentration in the IM (SMD: 0.24; 95% CI, 0.00, 0.47; *P* = .05) compared with placebo and people with low-fiber intake.^85^ In general, plant products are associated with a positive effect on the IM. Vegetable protein consumption is associated with an increase in *Bifidobacterium *spp. and *Lactobacillus *spp. presence, as well as an increase in the concentration of short-chain fatty acids (SCFAs) in the IM and anti-inflammatory effects.^86,87^ The impact of IM correction through dietary interventions on the development and course of UTIs is not well understood. Cranberry-based products are widely used in combination therapy and prevention of UTIs.^88^ It is assumed that the therapeutic effect is based on the anti-adhesive activity of cranberry preparations and their ability to inhibit uropathogenic *E.* *coli*.^89,90^ The metabolism of cranberry complex carbohydrates called xyloglucans has been reported to stimulate the growth of “beneficial” intestinal bacteria such as *B. longum.* ^91^ Daily intake of cranberry juice for 24 weeks in women with a history of prior UTIs reduced the number of relapses to 39 episodes, compared with 67 episodes in the placebo group (*P* = .016). When studying the IM of study participants, it was found that one species of *Flavonifractor *(OTU41), which accounted for ≤1% of the total metagenome, was significantly less common in the group drinking cranberry juice. The species is reported to have genes related to the transport and metabolism of various compounds, including tryptophan and cobalamin, which play a role in host–microbe interactions.^92^ The process of tryptophan metabolism is involved in adaptive immune regulation and pathogenesis of epithelial infection with uropathogenic *E. coli.* ^93^ There was a reduction in recurrent UTIs in the cranberry and propolis group compared with placebo (0.7 vs. 1.3, *P* = .02), in addition, the mean time to the onset of the first UTI episode was significantly longer in the “propolis + cranberry” group (70 vs. 43 days, *P* = .03).^94^ A study of the vegan diet effect on the risk of developing UTIs in 9724 people found that a vegan diet was associated with a 16% lower risk of UTIs than a nonvegetarian diet. Marked risk reduction has been noted in women with uncomplicated UTIs.^95^ Vegans and vegetarians are known to have lower total numbers of *E. coli *and *Enterobacteriaceae *spp. in the IM composition and vice versa—more *Roseburia* and* Prevotella* than in nonvegetarians.^96,97^ *Prevotella* representatives are generally considered useful commensals due to their abundance in healthy individuals and their rare involvement in infectious and inflammatory processes. *Prevotella *spp. are associated with active propionate production.^98^ It has been found that the genome associated with increased virulence, as well as antibiotic resistance and possible pro-inflammatory *Prevotella* properties, is significantly more common in Western populations than in populations whose diets are traditionally based on plant products.^99^ Polyphenols, which are also abundant in plant products, increase the number of *Bifidobacterium *spp. and* Lactobacillus *spp., which have anti-inflammatory effects.^100^ However, due to a small number of studies and heterogeneity and limitations in different studies, further study of the effect of dietary interventions on IM and UTI is required.

## Conclusion

The results of numerous studies confirm the need to take into account the IM influence on the development of UTIs. Intestinal microbiota correction can become a promising method in disease treatment and prevention. Positive results have been shown for pro/prebiotics, FMT, and dietary interventions. However, at the moment, there is not enough data to form reliable recommendations on the use of such correction methods in the treatment and prevention of UTIs, which determines the feasibility of further research in this direction.

## Figures and Tables

**Table 1.** Current Evidence on Antimicrobial Treatment and Prevention of Urinary Tract Infections

Studies	Results
Trimethoprim-sulfamethoxazole (cotrimoxazole)	
Vachhani et al^28^	The bacteriological cure rate with 3 days of treatment was 86.2%.
Rezaei-Tavirani et al^29^	The prevalence of resistance was: *E. coli*—62%, *Klebsiella*—54%, *Staphylococcus*—55%, *Enterobacter*—52%.
Crellin et al^30^	Trimethoprim increased the risk of acute kidney injury and hyperkalemia compared to amoxicillin.
Wesolek et al^31^	25.1% of *E. coli*-positive urine samples were resistant to cotrimoxazole.
Drekonja et al^32^	There was no statistically significant difference in the treatment with trimethoprim-sulfamethoxazole compared with ciprofloxacin.
Nitrofurantoin	
Huttner et al^33^	Clinical resolution was achieved in 70% of patients in the nitrofurantoin group (5 days) compared to 58% of patients in the fosfomycin group (single dose).
Huttner et al^34^	Clinical cure rates were 79%-92%. Overall equivalence in clinical cure between nitrofurantoin and trimethoprim-sulfamethoxazole, ciprofloxacin, and amoxicillin has been reported. Toxicity was infrequent (5%-16%) and predominantly gastrointestinal (nausea, vomiting, abdominal pain, and diarrhea).
Porreca et al^35^	The clinical cure rates in nitrofurantoin ranged from 51% to 94%, and bacteriological cure rates ranged from 61% to 92%. Gastrointestinal and central nervous system symptoms were reported.
Mitrani-Gold et al^36^	The overall microbiological response was 0.766 (0.665-0.867) for nitrofurantoin and 0.342 (0.288-0.397) for placebo.
Konwar et al^37^	Multiple studies have found that nitrofurantoin (100 mg for 5 days) is more effective than other first-line drugs.
Fosfomycin	
Wang et al^38^	Fosfomycin (single dose) was comparable to other antibiotics in terms of clinical and microbiological resolution of UTIs. The most common adverse events were mainly gastrointestinal.
Cai et al^39^	No differences were found in microbiological eradication and clinical resolution with fosfomycin compared with other antibiotics. Single dose is associated with high patient adherence.
Fajfr et al^40^	The resistance to fosfomycin in *Escherichia coli* isolates before and after registration of the drug did not differ significantly (3.4% and 4.4%, respectively). In some other gram-negative rods, such as otherwise sensitive enterobacteria, fosfomycin resistance increased significantly from 45.6% to 76.6%. In the treatment of recurrent or complicated urinary tract infections, fosfomycin treatment was associated with a high rate of recurrence of the infection (20.4% during the first 2 months).
Ten Doesschate et al^41^	In patients treated with fosfomycin, microbiological cure occurred in 29 of 37 (78.4%). Gastrointestinal adverse events were reported in 25 of 48 (52.1%).
Fluoroquinolones	
Langner et al^42^	The most commonly prescribed antibiotics for the treatment of UTIs from 2015 to 2019 were fluoroquinolones (36.4%; 16.3 million visits of 44.9 million visits).
Cowart et al^43^	FDA warning did not significantly affect fluoroquinolone prescribing rates.
Beta-lactams	
Anesi et al^44^	Extended-spectrum cephalosporin resistance was associated with an increased hazard of recurrent UTI.
Bunduki et al^45^	Resistance UPEC isolates to first-generation cephalosporins was 38.8% (370/953).
Mortazavi-Tabatabaei SAR et al^46^	The resistance among the isolates of *E. coli* was as follows: ampicillin (86%), amoxicillin (76%), cephalexin (61%), and cefalotin (60%).

FDA, Food and Drug Administration; UPEC, uropathogenic *Escherichia coli*; UTIs, urinary tract infections.

**Table 2.** Non-Antimicrobial Methods of Treatment and Prevention of Urinary Tract Infections

Studies	Results
Phytotherapy, herbal treatment, and nutraceutical compounds	
Cai et al^53^	A medical device containing xyloglucan, hibiscus, and propolis is superior to placebo in clinical efficacy and is associated with high patient compliance.
Gágyor et al^54^	Treatment with uva ursi extract reduced antibiotic use was 63.6% lower (95% CI 53.6-71.4%; *P* < .0001) compared to fosfomycin group. A higher incidence of pyelonephritis was reported in the uva ursi-group.
Wagenlehner et al^55^	The use of BNO 1045 is not inferior to fosfomycin in the treatment of UTIs, with fewer gastrointestinal side effects and a higher incidence of pyelonephritis reported compared to fosfomycin.
Cranberry	
Xia et al^56^	Consumption of cranberry-based products can significantly reduce the incidence of UTIs (RR = 0.70; 95% CI, 0.59-0.83; *P* < .01). A relative risk reduction of 32% has been reported in women with recurrent UTIs (RR = 0.68; 95% CI, 0.56-0.81).
d-mannose	
Kyriakides et al^57^	Taking d-mannose increased the time to recurrence and improved the quality of life.
Vaccines	
Prattley et al^58^	The short-term role of vaccination in the prevention of recurrent urinary tract infections has been reported.
Methenamine hippurate	
Harding et al^59^	Methenamine hippurate showed comparable results in the prevention of UTIs at 12 months of treatment compared with antibiotics
Bakhit et al^60^	A nonstatistically significant trend of benefits for methenamine hippurate for the number of UTIs was found.
Harding et al^61^	Methenamine hippurate is not inferior to daily low-dose antibiotics in preventing recurrent UTIs.
Nonsteroidal anti-inflammatory drugs	
Ong Lopez et al^62^	NSAID therapy has less clinical and microbiological efficacy and a higher incidence of UTI complications compared to antibiotics.
Probiotics	
New et al^63^	Probiotics may reduce the risk of UTIs and have a limited side-effect profile.

NSAID, nonsteroidal anti-inflammatory drugs; RR, risk ratio; UTIs, urinary tract infections.
